# Prediction and analysis of toxic and side effects of tigecycline based on deep learning

**DOI:** 10.3389/fmicb.2024.1512091

**Published:** 2024-12-17

**Authors:** Yin Xiong, Guoxin Liu, Xin Tang, Boyang Xia, Yalian Yu, Guangjun Fan

**Affiliations:** ^1^Department of Pharmacy, The Second Affiliated Hospital of Dalian Medical University, Dalian, Liaoning, China; ^2^Department of Intervention, Shengjing Hospital of China Medical University, Shenyang, Liaoning, China; ^3^Department of Orthopedics, The Second Hospital of Dalian Medical University, Dalian, Liaoning, China; ^4^School of Government and Public Affairs, Communication University of China, Beijing, China; ^5^Department of Otorhinolaryngology, The First Affiliated Hospital of China Medical University, Shenyang, Liaoning, China

**Keywords:** deep learning, tigecycline, pharmacokins, hospital days, hepatotoxicity

## Abstract

**Background:**

In recent years, with the increase of antibiotic resistance, tigecycline has attracted much attention as a new broad-spectrum glycylcycline antibiotic. It is widely used in the treatment of complex skin and soft tissue infections, complex abdominal infections and hospital-acquired pneumonia by inhibiting bacterial protein synthesis. Tigecycline can exhibit significant time-dependent bactericidal activity, and its efficacy is closely related to pharmacokinetics. It can be evaluated by the ratio of AUC0-24 to the minimum inhibitory concentration (MIC) of pathogens. However, tigecycline may cause nausea, vomiting, diarrhea and a few patients have elevated serum aminotransferase, especially in critically ill patients. The safety of patients still needs further study.

**Methods:**

In this study, the clinical data of 263 patients with pulmonary infection in Shengjing Hospital of China Medical University and the Second Affiliated Hospital of Dalian Medical University were collected retrospectively, and the hepatotoxicity prediction model was established. The potential correlation between the toxic and side effects of tigecycline and the number of hospitalization days was preliminarily discussed, and the correlation analysis between the number of hospitalization days and continuous variables was established. Finally, the deep learning model was used to predict the hospitalization days of patients through simulated blood drug concentration and clinical laboratory indicators.

**Results:**

The degree of abnormal liver function was significantly correlated with AST, GGT, MCHC and hospitalization days. Secondly, the correlation between hospitalization time and clinical test indexes and simulated drug concentration was analyzed. It was found that multiple clinical laboratory parameters of patients (such as EO #, HCT, HGB, MCHC, PCT, PLT, WBC, AST, ALT, Urea), first dose (Dose), age and APACHE II score were significantly correlated with hospitalization days. The simulated blood drug concentration was correlated with the length of hospital stay from 12 h after administration, and reached the strongest between 24 and 48 h. The AUC of the liver function prediction model can reach 0.90. Further analysis showed that there was a potential correlation between hepatotoxicity and hospitalization days. The median hospitalization days of patients in the non-hepatotoxicity group, liver function injury group and hepatotoxicity group were 20, 23, and 30 days, respectively. Based on these results, the length of hospital stay was predicted by the deep learning prediction model with an error within 1 day.

**Conclusion:**

In this study, the hospitalization days of infected patients were predicted by deep learning model with low error. It was found that it was related to clinical test parameters, hepatotoxicity and dosage after administration. The results provided an important reference for the clinical application of tigecycline, and emphasized the need to pay attention to its toxic and side effects in use.

## 1 Introduction

In recent years, with the increasing resistance of antibiotics, the treatment of infection has become more complicated, which further highlights the necessity of new antibiotics in clinical application (Gupta et al., 2017).

Tigecycline, as a new broad-spectrum glycylcycline antibiotic, plays a role by inhibiting bacterial protein synthesis. It is widely used in the treatment of complex skin and soft tissue infections, complex abdominal infections, and hospital-acquired pneumonia and other infections (Bradford et al., 2005; Xie et al., 2017). First, tigecycline has obvious time-dependent bactericidal activity, and the efficacy is closely related to the relationship between pharmacokinetics (PK) and pharmacodynamics (PD). The ratio of AUC0-24 to the minimum inhibitory concentration (MIC) of the pathogen can better predict the therapeutic effect of the drug (Van Wart et al., 2006; Koomanachai et al., 2009; Bhavnani et al., 2012). Tigecycline is mainly excreted through bile, and its excretion in the kidney is low, only about 20% of the prototype drug, which provides more options for the use of patients with renal insufficiency (Ap, 2008; Yamashita et al., 2014).

The most common adverse reactions of tigecycline in clinical application are nausea, vomiting and diarrhea, but in phase 2 and phase 3 clinical trials, it was found that about 2–5% of patients had elevated serum aminotransferase (Babinchak et al., 2005; Ellis-Grosse et al., 2005; Sacchidanand et al., 2005). Elevated serum aminotransferase often suggests abnormal changes in liver function. However, so far (Geng et al., 2018), in the field of related research, there are few studies on the abnormal liver function caused by the use of tigecycline in critically ill patients. This may hinder the comprehensive understanding of the safety characteristics of tigecycline, and the use of tigecycline may cause serious damage to the liver function of critically ill patients, thus affecting the therapeutic effect of patients.

Population pharmacokinetic model (PPK) is a mathematical model that can describe the typical pharmacokinetic characteristics and variability of the population by integrating the plasma concentration and individual information of multiple individuals and considering the variability between individuals and within individuals. The model can effectively capture the influence of covariates such as patient’s age, weight, and disease status on pharmacokinetic parameters. Through the combination of PPK model and Bayesian method, compared with the traditional analysis method, the advantage of Bayesian theorem is that it can make full use of prior information and improve the accuracy of estimation. Through the dynamic feedback mechanism, the continuous optimization of model parameters is realized, which is effectively applied to complex and changeable situations, so as to accurately simulate the blood concentration of individual patients.

In recent years, artificial intelligence technology (Iezzi et al., 2019) has gradually shown broad application prospects in pharmacokinetic studies. Based on Deep Learning (LeCun et al., 2015), it can not only process large-scale biomedical data, but also identify complex nonlinear relationships. This ability makes AI a powerful tool in pharmacokinetic studies, especially in the fields of drug concentration prediction, drug interaction analysis, and risk assessment of adverse reactions, thereby providing support for personalized medication and clinical decision-making. A 2023 study explored the significant development of therapeutic drug monitoring (TDM) and model-guided precision drug delivery (MIPD) driven by advances in computing and mathematical technology (Poweleit et al., 2023). A 2022 study that combines a physiologically based pharmacokinetic (PBPK) model with machine learning (ML) or artificial intelligence (AI) techniques to predict ADME parameters using ML/AI, and integrates these prediction models into the PBPK model to predict pharmacokinetic (PK) statistical results (Chou and Lin, 2023). In another study, neural-ODE is applied to PK modeling for the first time. The final results show that it has a wide range of applicability and may have an important impact on future research (Lu et al., 2021). All of these indicate the potential application of artificial intelligence in pharmacokinetic analysis.

Therefore, this study aims to explore the potential risk factors for hepatotoxicity in patients treated with tigecycline by means of artificial intelligence-based technology. By analyzing the correlation between abnormal liver function and laboratory parameters, hospitalization days, etc., the mechanism of hepatotoxicity of tigecycline and its potential relationship with prolonged hospitalization days were revealed.

In addition, by exploring the effect of tigecycline hepatotoxicity on hospitalization days, a prediction model of hospitalization days was established to provide scientific reference for clinical practice, and then provide an important reference for optimizing the clinical application of tigecycline.

## 2 Method

### 2.1 Study population

**Figure 1:**
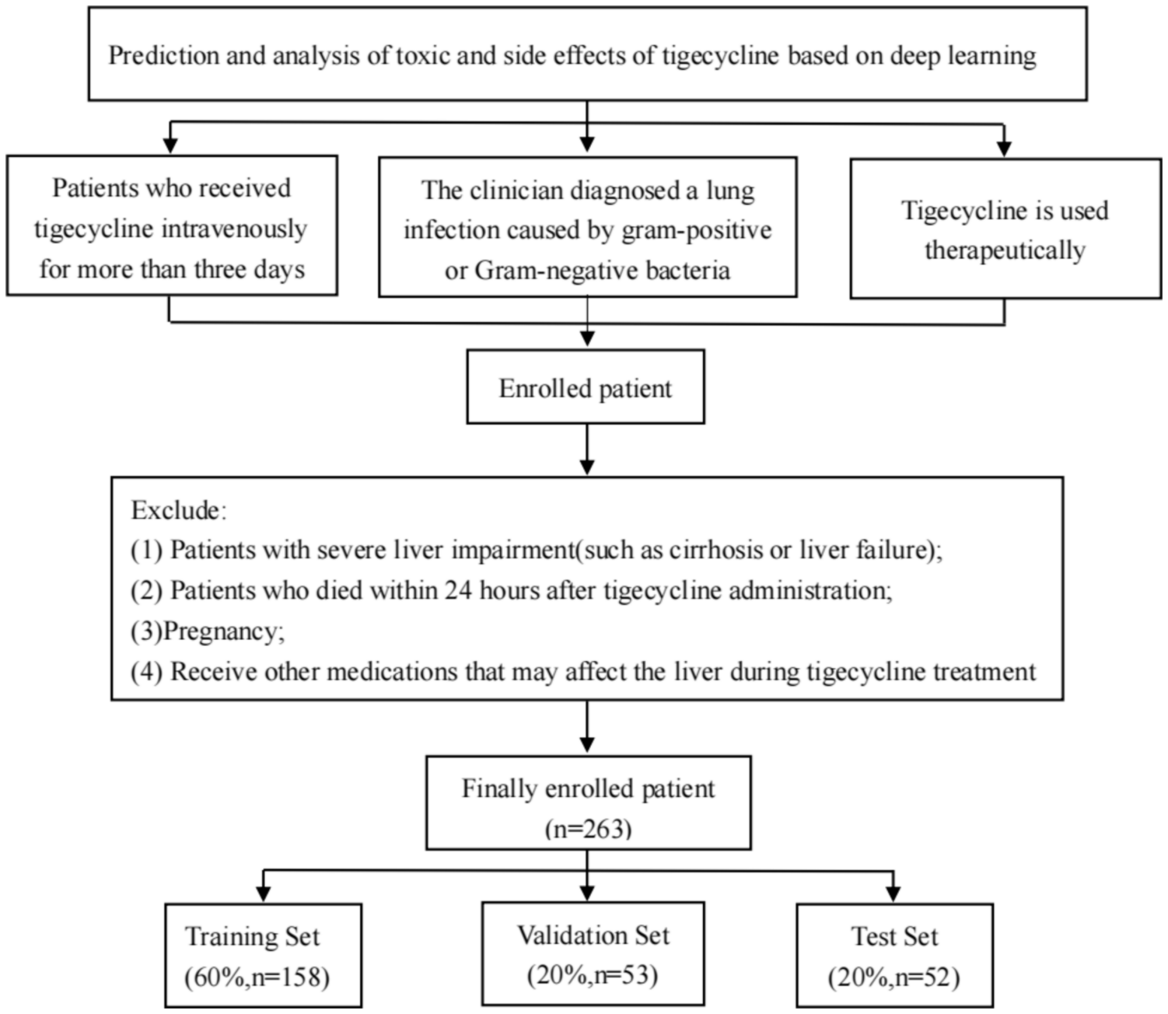
Inclusion and exclusion process of patients in this study. Finally, 263 patients using tigecycline in two centers were retrospectively collected through the inclusion and exclusion criteria, and the data were trained in a deep learning model according to the corresponding proportion.

This study retrospectively collected the clinical data of two patients with cardiopulmonary infection. The study was approved by the Ethics Committee, Ethics No. (2019 no. 049). Inclusion criteria: Patients included in this study should meet the following criteria: (1) patients with clinical intravenous use of tigecycline for more than three days; (2) Tigecycline is for therapeutic use; (3) Pulmonary infection caused by Gram-positive or Gram-negative bacteria, such as pneumonia or bronchitis, is diagnosed or highly suspected by clinicians. Exclusion criteria: (1) patients with cirrhosis or liver failure; (2) Patients died within 24 h after the use of tigecycline; (3) Pregnancy; and (4) Other medications that may affect the liver during tigecycline treatment. Inclusion and exclusion as shown in Figure 1.

### 2.2 Data collection

All enrolled patients were treated with tigecycline, and laboratory indicators and basic information were recorded in detail. This study collected patient data through the hospital’s electronic medical record system and nursing system. The collected laboratory indicators included: (1) patient age, initial dose of tigecycline (Dose), and length of hospital stay; (2) laboratory examination results of patients during medication. Such as EO # (absolute number of eosinophils), EO % (percentage of eosinophils), HCT (hematocrit), HGB (hemoglobin), MCH (mean corpuscular hemoglobin content), MCHC (mean corpuscular hemoglobin concentration), MCV (mean corpuscular volume), MPV (mean platelet volume), PCT (platelet hematocrit), PLT (platelet count), WBC (white blood cell count), RH (hemorheology), A/G ratio (albumin/globulin ratio), ALT (alanine aminotransferase), AST (aspartate aminotransferase), GGT (*γ*-glutamyl transferase), TP (total protein), Urea (urea), and APACHE II score. (3) Drug-induced adverse reactions, such as hepatotoxicity.

The abnormal liver function of tigecycline was defined as the ALT value measured twice in a row was between the upper limit of the normal value (5–40 U/L) and the upper limit of the normal value by 3 times, and its hepatotoxicity was defined as the ALT value measured twice in a row >3 times the upper limit of the normal value (or blood bilirubin >1.5 times the upper limit of the normal value), or greater than 1.5 times the baseline value (if the baseline value is abnormal) (Fan et al., 2020).

### 2.3 Blood concentration simulation

In this study, we constructed a population pharmacokinetic model based on previous research results (Luo et al., 2023) (Equations 1, 2). Bayesian feedback method (Aggelopoulos, 2015) was used to simulate the blood concentration of patients at different time points after administration. We determined the mathematical form of the model, including key kinetic processes such as drug absorption, distribution, metabolism, and excretion, taking into account individual differences, residual variation, and drug characteristics. The parameter values of the model were based on previous research reports, and the Bayesian feedback method was used to simulate the corresponding blood concentration values at different time points. The final model formula:


(1) $$CL\left(L/h\right)=\left(11.30-0.14*\mathrm{APACHE}\;II\right)*e^{0.065}$$

(2) $$V\left(L\right)=\left[105.00*\left(1-0.0059*\mathrm{AGE}\right)\right]*e^{0.160}$$

### 2.4 Deep learning model

**Figure 2:**
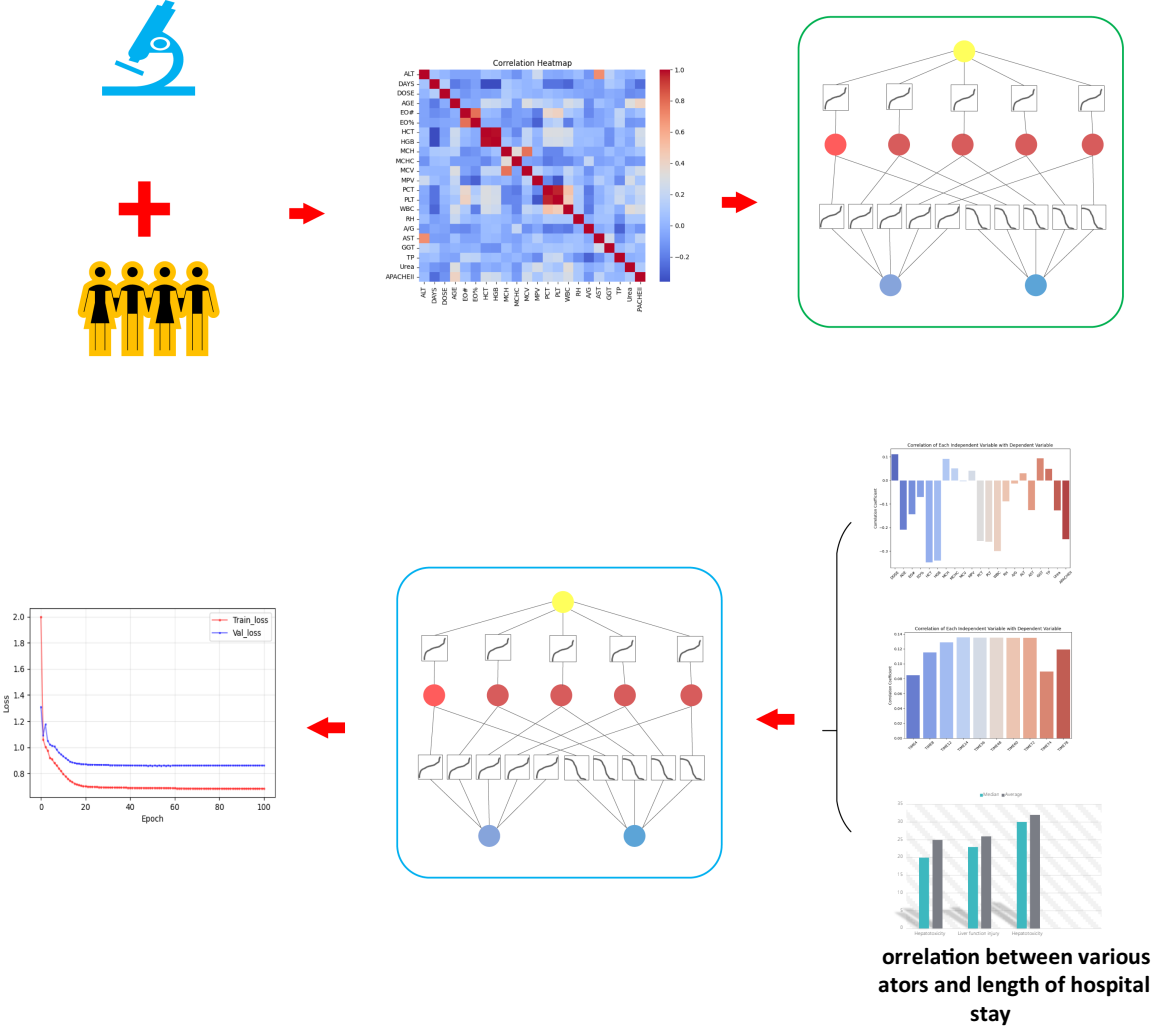
Overall flow chart of this study. **(A)** This study retrospectively collected the clinical laboratory indicators of the two centers, and the blood concentration simulated by Bayesian feedback method, established the correlation between liver function and clinical test, and based on this, a hepatotoxicity prediction model was constructed. **(B)** Establish the correlation between hospitalization days and clinical experimental indicators and simulated blood concentration, and explore and confirm the potential relationship between hospitalization days and hepatotoxicity. By inputting test indicators and blood concentration to the KAN network, the hospitalization days were predicted and the hepatotoxicity was indirectly predicted, and finally the prediction results with lower errors were obtained.

In order to solve the complex problem of hepatotoxicity prediction during treatment, we used the latest KAN network (Liu et al., 2024) to establish a tigecycline hepatotoxicity prediction model. KAN is derived from Kolmogorov-Arnold representation theorem, whose core idea is that any continuous function can be represented by a combination of one-dimensional functions. We map the blood drug concentration and the patient’s clinical laboratory indicators to the input layer of the KAN model. Through its specific multi-layer structure, KAN nests these multi-dimensional input features into a one-dimensional function combination, and then approximates the complex nonlinear relationship between drugs and liver function indicators. Based on these input characteristics, we established a predictive model that can not only predict the number of days of hospitalization, but also further predict the possible liver toxicity of patients during treatment, and provide support for personalized treatment and clinical decision-making. The research flow chart of this study is shown in Figure 2.

### 2.5 Statistical analysis

Statistical analysis was performed using IBM SPSS 26.0 software. First, we test the normality of all data using the Kolmogorov–Smirnov test. For data that conform to the normal distribution, we use the mean ± standard deviation to describe; for data that do not conform to the normal distribution, the median (quartile) is used to describe. For categorical data, the t test was used for data that conformed to the normal distribution, and the Mann–Whitney U test was used for data that did not conform to the normal distribution. For continuous variables, Pearson correlation analysis was used for data that met the normal distribution, and Spearman correlation analysis was used for data that did not meet the normal distribution. Differences were considered statistically significant at *p* < 0.05. The statistics chart will be drawn using the Python-based matplotlib library.

## 3 Results

### 3.1 Baseline

A total of 263 patients from two centers were included in this study: 200 from Institution I (Shengjing Hospital Affiliated to China Medical University) and 63 from Institution II (Second Hospital Affiliated to Dalian Medical University). All patients were treated with tigecycline. In terms of data, the training, validation and test sets are divided according to the proportion of 6: 2: 2 patients, which ensures the rationality of model training, tuning and evaluation. This division method helps to improve the generalization ability of the model and effectively avoid over-fitting. Table 1 lists the baseline information of the included patients.

**Table 1:** Patient baseline information.

Hospital name	Total number of patients	Number of participants in the training set (60%)	Number of people in the validation set (20%)	Number of people in the test set (20%)	Average age (year)	Age range (year)
Institution I	200	120	40	40	66	14–98
Institution II	63	38	13	12	58	18–89
Total	263	158	53	52	62	14–98

### 3.2 Abnormal liver function, toxicity and laboratory relevance

Different laboratory indicators are helpful to evaluate the degree of liver injury. The overall ALT group, abnormal liver function group and hepatotoxicity group were established to explore the relationship between these three groups and laboratory indicators. Based on this grouping, the effects of different types and degrees of liver injury on various test indicators can be accurately analyzed. As shown in Table 2, it was found that there was a significant correlation between the number of hospital stays and the ALT group and the abnormal liver function group, suggesting that liver function damage may affect the length of hospital stay. At the same time, MCHC, AST and GGT showed significant correlation with the three groups, indicating that poor liver function can indirectly affect the formation and function of red blood cells, thus affecting the MCHC value. In addition, liver cells are damaged, and AST is released from the liver cells into the blood, resulting in an increase in AST, and if the biliary system is damaged, GGT levels will increase accordingly.

**Table 2:** Abnormal liver function, toxicity and laboratory relevance.

Characteristic	DAYS	AGE	EO#	EO%	HCT	HGB	MCH	MCHC	MCV	MPV	PCT	PLT	WBC	RH	A/G	AST	GGT	TP	Urea	APACHE II
ALT	0.027^*^	0.950	0.687	0.702	0.265	0.254	0.650	0.027^*^	0.435	0.210	0.836	0.719	0.826	0.035^*^	0.169	0.001^*^	0.001^*^	0.389	0.643	0.690
Abnormal liver function	0.043^*^	0.661	0.037^*^	0.111	0.874	0.918	0.889	0.016^*^	0.216	0.610	0.740	0.975	0.950	0.437	0.041^*^	0.001^*^	0.001^*^	0.724	0.164	0.843
Hepatotoxicity	0.073	0.813	0.649	0.075	0.417	0.557	0.054	0.005^*^	0.601	0.001^*^	0.074	0.851	0.243	0.768	0.118	0.001^*^	0.048^*^	0.777	0.106	0.212

* The difference was statistically significant (*p* < 0.05).

### 3.3 Prediction of hepatotoxicity based on deep learning

**Figure 3:**
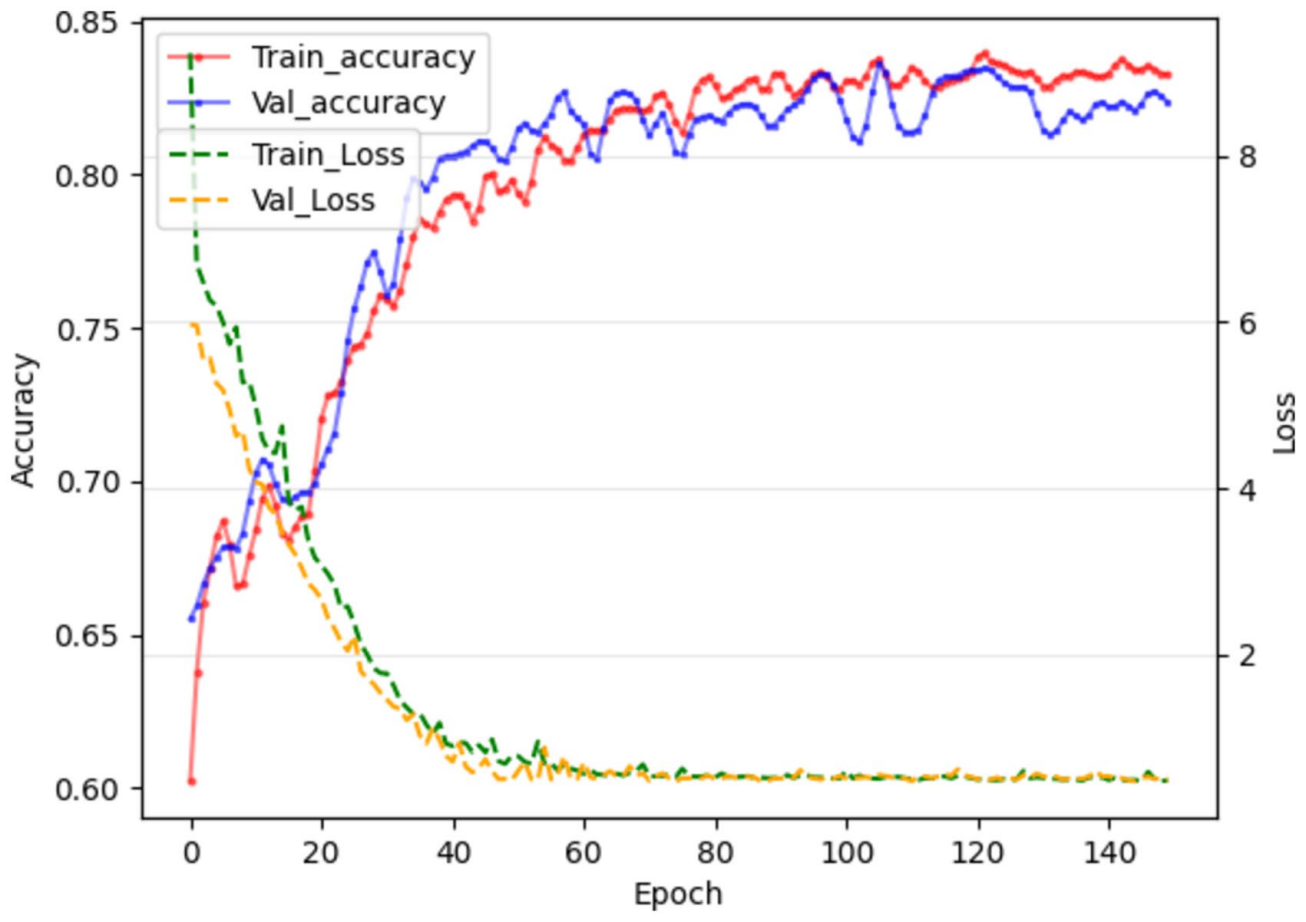
In the 121st round, the training and validation phases achieved the best results, and the accuracy of the liver function prediction model reached 84.71 and 83.76, respectively.

**Figure 4:**
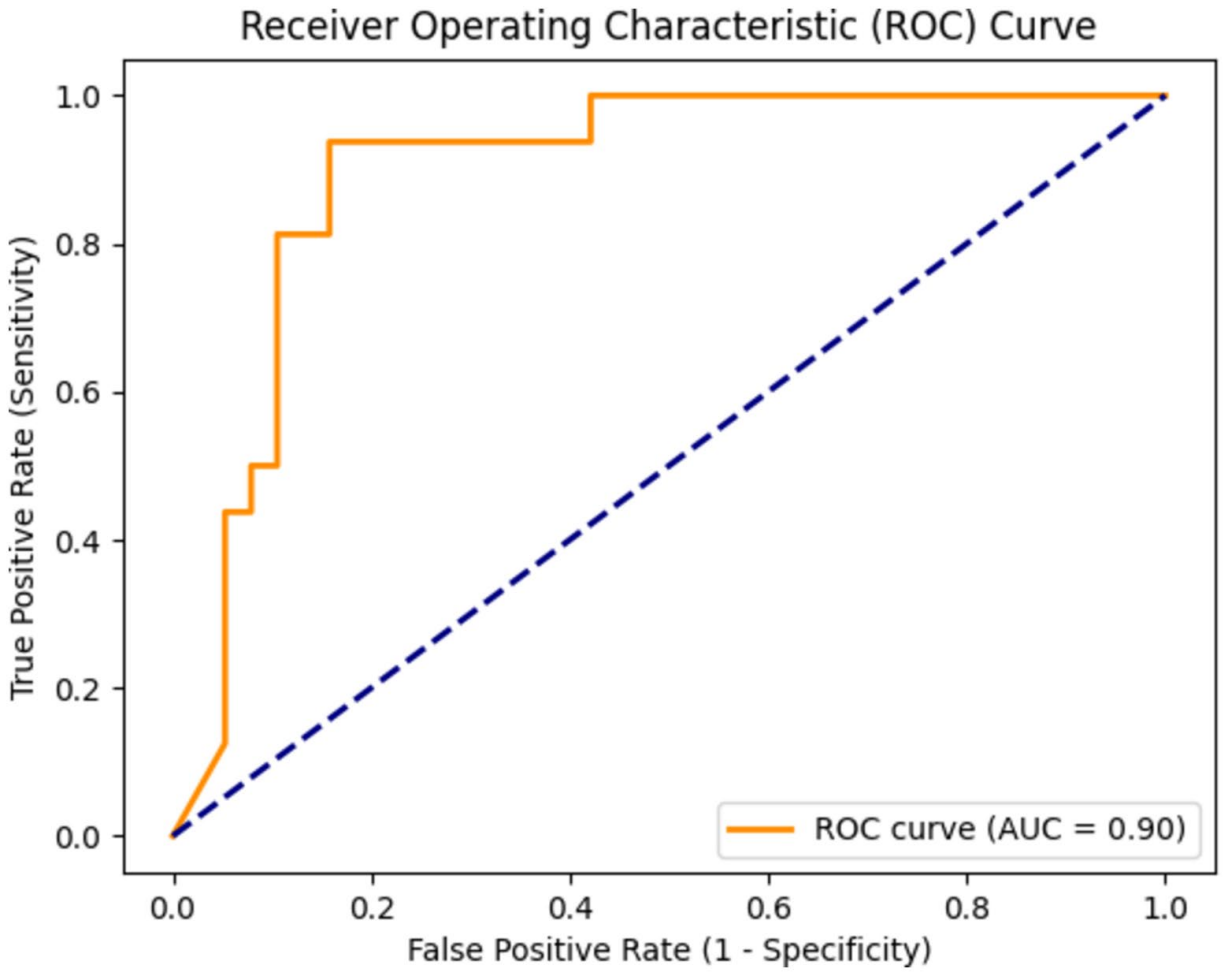
The area under the curve (AUC) was 0.90, indicating that the model had a high ability to distinguish whether there was abnormal liver function.

Due to the small sample size of patients with hepatotoxicity in this study, there are some challenges in directly predicting hepatotoxicity. Therefore, we indirectly reflect the potential risk of tigecycline-induced hepatotoxicity by predicting whether the patient has abnormal liver function. The training results are shown in Figure 3. The model showed high performance in the testing set, with a sensitivity of 0.94, a specificity of 0.87, and an AUC value of 0.9, as shown in Figure 4. This shows that by predicting the abnormal liver function of hospitalized patients, we can indirectly capture the potential risk of hepatotoxicity and provide an early warning model for early clinical identification and intervention of possible liver injury.

### 3.4 Potential association between liver function and length of hospital stay

**Figure 5:**
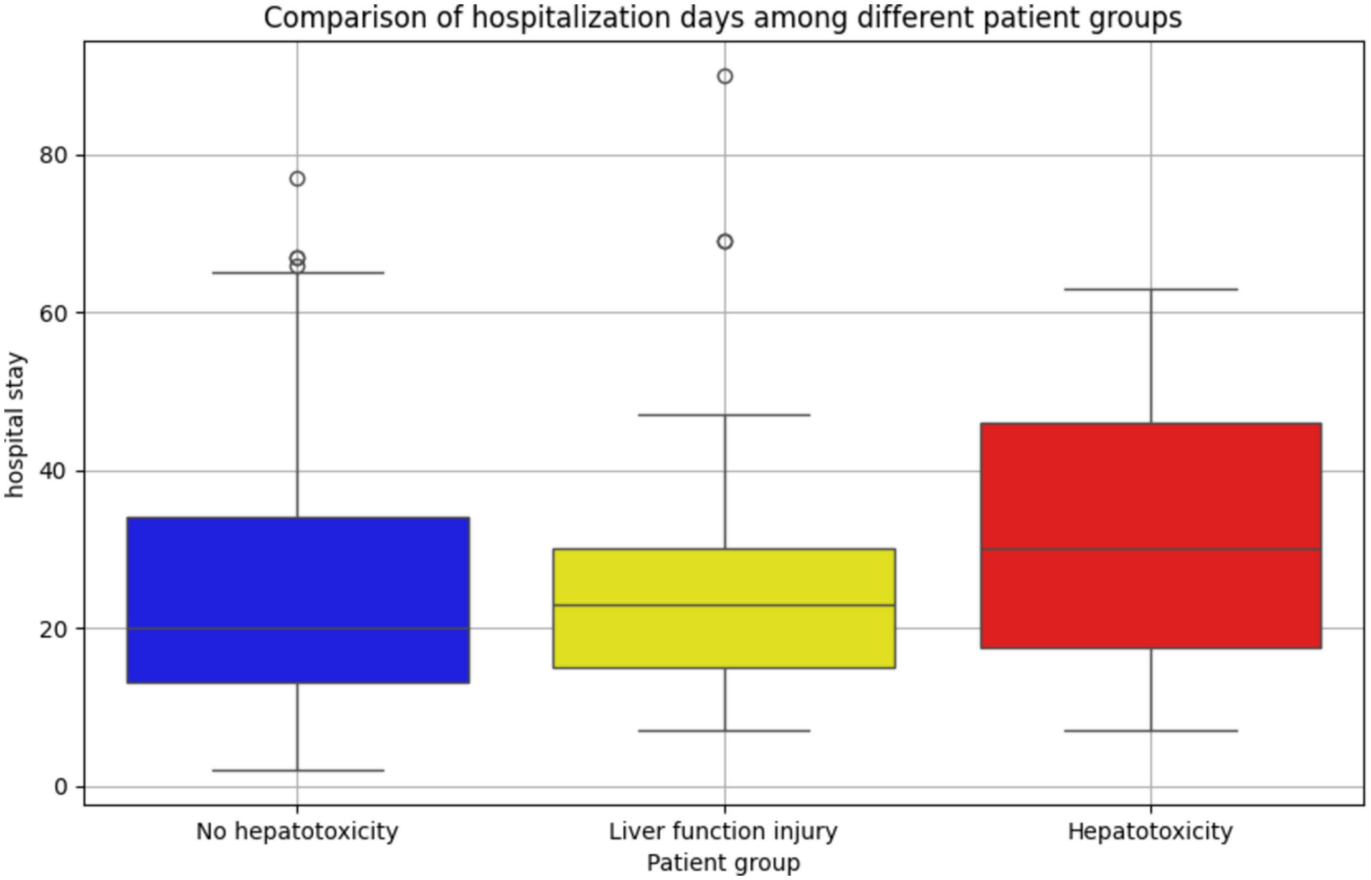
Distribution and difference of hospitalization days in patients with different liver function status. The number of hospitalization days in the non-hepatotoxicity group was the most widely distributed, and there were more extreme hospitalization days. The distribution of hospitalization days in the liver function injury group was more concentrated, and the hospitalization time was longer as a whole; the distribution of hospitalization days in the hepatotoxic group was more balanced, and no abnormal value was found. It reflects the difference in hospitalization days among different patient groups.

Although we cannot directly analyze the relationship between drug-induced hepatotoxicity and length of hospital stay, we can preliminarily explore whether there is a potential correlation between the degree of abnormal liver function after drug treatment and the length of hospital stay. Therefore, we divided the patients into three groups according to the definition of hepatotoxicity as shown in Table 3: hepatotoxic group, liver function injury group (defined as two consecutive ALT values between 40 U/L and 120 U/L), and non-hepatotoxic group. Through Figure 5, the box diagram visually shows the distribution characteristics and abnormal values of hospitalization days in different patient groups. The results showed that the length of hospital stay in the non-hepatotoxic group was shorter, with a median of 20 days, but the distribution of length of hospital stay was wider, suggesting that there may be extreme values of individual length of hospital stay in this group. For the liver function injury group, the distribution of hospitalization days was relatively concentrated and the variability was small. The median (23 days) was located above the box, indicating that the hospitalization days of this group of patients were generally longer. Finally, the box of the hepatotoxicity group was wider and the overall was higher, indicating that the median length of hospital stay in this group was higher (30 days), and the overall length of hospital stay was the longest among the three groups. In addition, there was no significant extreme value or outlier in the length of hospital stay in this group. The overall results reflect the significant differences in the number of hospitalization days among different patient groups, suggesting that hepatotoxicity may have a greater impact on the length of hospitalization, while the liver function injury group showed a more uniform hospitalization demand.

**Table 3:** Descriptive statistics of the number of hospitalization days in the patient group.

Patient group	Number of people	Average length of hospital stay	Median length of hospital stay	Standard deviation
No hepatotoxicity	202	25	20	16.37
Liver function injury	50	26	23	16.59
Hepatotoxicity	11	32	30	19.97

The difference was statistically significant (*p* < 0.05).

### 3.5 Correlation between hospitalization days and clinical laboratory indexes

Table 4 shows the correlation between the length of hospital stay and clinical information of the included patients. Among the laboratory parameters, EO#, HCT, HGB, MCHC, PCT, PLT, WBC, AST, ALT, urea *p* < 0.05, that is, these indicators may be potential factors reflecting hepatotoxicity. The *p* value of dose was <0.05, indicating that higher doses may lead to greater toxic and side effects, which in turn leads to longer hospital stay. Similarly, age and APACHEII *p* values <0.05 indicated that tigecycline had a higher risk of toxicity in elderly or more severe patients.

**Table 4:** The correlation between continuous variables and hospitalization days.

Indicator	Statistic	*p*-value	Indicator	Statistic	*p*-value
Age	263	0.001^*^	WBC	263	0.001^*^
EO#	263	0.004^*^	Dose	263	0.048^*^
EO%	263	0.065	RH	263	0.053
HCT	263	0.001^*^	A/G	263	0.345
HGB	263	0.001^*^	ALT	263	0.027^*^
MCH	263	0.092	AST	263	0.001^*^
MCHC	263	0.008^*^	GGT	263	0.367
MCV	263	0.161	TP	263	0.151
MPV	263	0.323	Urea	263	0.048^*^
PCT	263	0.001^*^	APACHEII	263	0.001^*^
PLT	263	0.001^*^			

* The difference was statistically significant (*p* < 0.05).

### 3.6 The correlation between hospitalization days and blood drug concentration

The plasma concentration (ng/ml) of patients at 10 time points (4, 8, 12, 24, 36, 48, 60, 72, 74, and 78 h) after the first administration was simulated by the constructed pharmacokinetic model. The results are shown in Table 5. In the correlation analysis of hospitalization days and blood drug concentration, as shown in Table 6, the correlation began to appear 12 h after administration, and reached the strongest between 24 and 48 h. To a certain extent, this shows that the residual concentration of the drug is the most significant toxic and side effects for patients in the 24–48 h period. Therefore, it is recommended to use this time period as an important node for clinical testing so that clinicians can make corresponding intervention and treatment decisions in a timely manner.

**Table 5:** Through the constructed pharmacokinetic model, the blood concentration at different time points after the first administration was simulated.

	Training set/158	Validation set/53	Testing set/52
4.0 h	1568.23 (728.41, 3119.10)	1605.29 (780.61, 2266.90)	1480.72 (762.71, 2380.40)
8.0 h	900.72 (432.17, 1881.60)	870.55 (426.39, 1413.80)	886.90 (449.74, 1582.30)
12.0 h	526.26 (216.02, 1425.90)	480.67 (194.17, 881.76)	522.87 (224.83, 1051.80)
24.0 h	371.16 (134.19, 1016.30)	325.21 (115.42, 654.79)	371.71 (140.44, 834.83)
36.0 h	340.65 (124.87, 929.15)	298.69 (107.98, 599.72)	340.84 (363.75, 771.1)
48.0 h	334.30 (123.61, 910.61)	293.87 (107.28, 586.37)	334.27 (128.61, 752.39)
60.0 h	332.91 (123.45, 906.67)	292.95 (107.21, 583.13)	332.81 (128.45, 746.89)
72.0 h	332.58 (123.43, 905.83)	292.76 (107.21, 582.34)	332.48 (128.42, 745.27)
74.0 h	1283.29 (633.02, 2562.00)	1298.10 (641.67, 1895.20)	1245.04 (654.06, 2067.50)
78.0 h	742.19 (349.25, 1578.20)	710.59 (348.88, 1182.00)	731.19 (363.74, 1374.30)

### 3.7 Prediction results of hospitalization days based on deep learning

**Figure 6:**
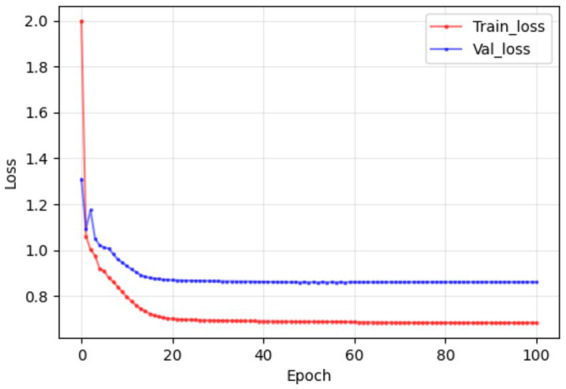
In the training and validation stages, the model maintained a good consistency in the prediction of hospitalization days, and the error could eventually be maintained within 1 day, which confirmed that the model established in this study had good prediction performance.

The simulated blood drug concentration and laboratory parameters were integrated into the constructed deep learning model. The training results are shown in Figure 6. In the training and verification stages, we observed that the model loss was maintained below 1. This result means that the error between the predicted hospitalization days and the actual hospitalization days can be effectively controlled within one day, showing the high accuracy and reliability of the model in predicting hospitalization days (Table 6).

**Table 6:** Correlation between blood drug concentration and hospitalization days.

		4 h	8 h	12 h	24 h	36 h	48 h	60 h	72 h	74 h	78 h
Hospital days	Statistic	263	263	263	263	263	263	263	263	263	263
	*P*-value	0.193	0.111	0.029^*^	0.010^*^	0.010^*^	0.010^*^	0.012^*^	0.011^*^	0.201	0.110

* The difference was statistically significant (*p* < 0.05).

## 4 Discussion

This study first analyzed the effect of liver function injury on test indicators. The results showed that the length of hospital stay was significantly correlated with ALT group and abnormal liver function group, suggesting that liver function injury may prolong the length of hospital stay. At the same time, MCHC, AST and GGT were significantly correlated with the three groups, reflecting the impact of poor liver function on red blood cell function, liver cell damage and biliary system damage, and had potential clinical evaluation value.

Secondly, the liver function early warning model established in this study has good performance and indirectly captures the potential risks of hepatotoxicity. By exploring the potential relationship between hospitalization days and tigecycline hepatotoxicity, the results showed that the degree of liver function damage was associated with hospitalization days. The hospitalization time of the patients in the hepatotoxicity group was longer as a whole, and the hospitalization days of the patients in this group showed a large change, which may be related to the severity of hepatotoxicity and the increase of treatment needs.

Then, by analyzing the correlation between the actual hospitalization days and the patient information and clinical indicators, it was found that there was a significant correlation between a number of clinical indicators and the actual hospitalization days. The actual hospitalization days obtained by the analysis were strongly related to the patient’s age, WBC, EO #, Dose, HCT, HGB, AST, ALT, MCHC, Urea, PCT, PLT, APACHEII.

Finally, this experiment uses simple and easy-to-obtain hospitalization days to replace the clinical index values that need to be measured multiple times, and uses the constructed deep learning model to predict hospitalization days. The error between the predicted results and the actual days is controlled at about one day. However, this cannot completely replace the clinical judgment of the liver side effects of tigecycline. There is a certain error. As the patient ages, the body function and various indicators will gradually decline. According to the study of Huang et al., the in-hospital mortality and length of stay gradually increased with age (Huang et al., 2023), which was consistent with the results of this experiment. However, if we want to further explore the relationship between the liver side effects of tigecycline and age in this experiment, we need to further collect clinical index data for correlation test.

At the same time, it was also found that the liver side effects of tigecycline were negatively correlated with the WBC level of the patients. As the side effects increased, the WBC and PCT levels of the patients decreased. Li et al. found that cefoperazone combined with tigecycline in the treatment of ICU infection can effectively improve the therapeutic effect of the disease, and significantly enhance the bacterial clearance, while reducing serum WBC and PCT levels (Li and Zhang, 2022). This suggests that when tigecycline is used alone, although there is a certain effect on the infection of patients, the toxic and side effects also increase. Whether there is the possibility of cefoperazone alleviating the toxic and side effects of tigecycline, which also provides a direction for future research and provides evidence for this experiment; the toxic and side effects were negatively correlated with the levels of HCT, HGB, AST, MCHC, PCT and PLT. With the increase of toxic and side effects, the levels of HCT, HGB, AST, MCHC, PCT and PLT decreased, which represented the decrease of liver function and coagulation function.

In a number of case reports and experiments (Sabanis et al., 2015; McMahan and Moenster, 2017; Cui et al., 2019), it is mentioned that the use of tigecycline will affect the coagulation system of patients, cause coagulation disorders, and produce adverse clinical outcomes. Therefore, in this experiment, the use of simple and easy-to-obtain hospitalization days can be a good warning for the occurrence of adverse coagulation events in patients. At the same time, in the study of Zhang et al., it was also found that in patients using tigecycline, renal dysfunction also caused tigecycline-induced coagulation-related adverse events (Zhang et al., 2020). In this experiment, the toxic and side effects of patients were negatively correlated with urea levels. Urea showed the liver function of patients to a certain extent, which also provided more evidence sources for the clinical manifestations of tigecycline toxicity.

We can find that most clinical indicators point to the decline of liver function. In recent studies, the deep learning of pharmacokinetics is combined with clinical imaging to empower the metabolic changes of pharmacokinetics in various organs, and accurately segment and refine the role of organs (Arledge et al., 2022; Ota and Yamashita, 2022; Dhaliwal et al., 2024). Although this study replaced the toxic and side effects of tigecycline and analyzed the correlation with a number of laboratory indicators, the experiment can further carry out more accurate analysis in the metabolic imaging of the patient’s liver, match the number of days of hospitalization, and refer to the number of days of hospitalization. More accurate, and in the future, we can try to build a liver model to achieve a simulated drug metabolism process, provide clinicians with more predictive medication recommendations, and avoid excessive or excessive medication.

At the same time, there are still corresponding limitations in this experiment. In view of the lack of sample size of patients with hepatotoxicity in this study, although the liver function prediction model has alleviated the problem of insufficient number of patients with hepatotoxicity to a certain extent, it also limits the specific interpretation of drug hepatotoxicity prediction. At the same time, further research on the correlation between hepatotoxicity and hospitalization days needs to verify this conclusion in a larger patient group and other external centers in the future to enhance the reliability and generalization of the results. As well as the defects that the study did not include the total number of patients’ medications and the total amount of medications into the experimental data, the patient stratification was not diversified enough, resulting in the inability to present a more detailed correlation analysis, which will be improved in future research.

## 5 Conclusion

Tigecycline, as a new type of antibiotic, has shown good clinical application potential in the treatment of complex infections. This study preliminarily explored the correlation between drug liver dysfunction, toxicity and laboratory parameters. By indirectly reflecting the potential possibility of hepatotoxicity, an early warning model of liver injury that can be clinically identified and intervened early was established. The significant correlation between liver function status and hospitalization days, as well as hospitalization days and clinical laboratory parameters and simulated dose after the first administration was determined. Finally, through the prediction and analysis of drug side effects on the simple and easy-to-obtain hospitalization days, it provides an important reference for the clinical application of tigecycline, and suggests that the side effects of drugs should be paid attention to in clinical use, especially in critically ill patients.

## Data Availability

The raw data supporting the conclusions of this article will be made available by the authors, without undue reservation.
